# Retraction Note: Glioblastoma stem cell (GSC)-derived PD-L1-containing exosomes activates AMPK/ULK1 pathway mediated autophagy to increase temozolomide-resistance in glioblastoma

**DOI:** 10.1186/s13578-025-01528-1

**Published:** 2026-01-22

**Authors:** Yong Zheng, Liang Liu, Yan Wang, Shan Xiao, Rongkang Mai, Zifeng Zhu, Yiyao Cao

**Affiliations:** 1 Department of Neurosurgery, The Second Affiliated Hospital of Shenzhen University, (People’s Hospital of Shenzhen Baoan District), Longjing Second Road No. 118, Shenzhen, 518101 Guang Dong China; 2 Department of General Practice Medicine, The Second Affiliated Hospital of Shenzhen University (People’s Hospital of Shenzhen Baoan District), Longjing Second Road No. 118, Shenzhen, 518101 Guang Dong China; 3 Department of Endocrinology, The Second Affiliated Hospital of Shenzhen University, (People’s Hospital of Shenzhen Baoan District), Longjing Second Road No. 118, Shenzhen, 518101 Guang Dong China

Retraction:** Cell & Bioscience (2021) 11:63** 10.1186/s13578-021-00575-8

The Editor-in-Chief has retracted this article. Following the publication, multiple concerns regarding western blots, flow cytometry data and microscopy images presented in Figs. 1, 2, 4, 5 and 7, and 8 have been raised with previously published articles [1, 2]. The authors stated that a third party was involved in performing the experiments and providing the data.

The Editor-in-Chief therefore has lost confidence in the integrity of data presented in this article.

None of the authors has responded to any correspondence regarding this retraction wording.
